# A rare case of visceral leishmaniasis in an immunocompetent traveler returning to the United States from Europe

**DOI:** 10.1371/journal.pntd.0006727

**Published:** 2018-10-04

**Authors:** Lamia Haque, Merceditas Villanueva, Armand Russo, Youzhong Yuan, Eun-Ju Lee, Jeffrey Topal, Nikolai Podoltsev

**Affiliations:** 1 Department of Internal Medicine, Section of General Internal Medicine, Yale University School of Medicine, New Haven, Connecticut, United States of America; 2 Department of Internal Medicine, Section of Infectious Diseases, Yale University School of Medicine, New Haven, Connecticut, United States of America; 3 Department of Internal Medicine, Section of Hematology and Oncology, Yale University School of Medicine, New Haven, Connecticut, United States of America; 4 Department of Pathology, University of Arkansas for Medical Sciences, Little Rock, Arkansas, United States of America; 5 Department of Internal Medicine, Section of Hematology and Oncology, Weill Cornell Medicine, New York, New York, United States of America; Instituto de Ciências Biológicas, Universidade Federal de Minas Gerais, BRAZIL

## Abstract

A young, healthy traveler returning to the United States presented with fever, night sweats, splenomegaly, and pancytopenia. Bone marrow biopsy revealed leishmaniasis (*Leishmania infantum*), likely acquired in southern France. Although many cases of endemic visceral leishmaniasis (VL) have been reported in Europe, this is a rare case of imported VL in a healthy traveler returning from Europe to the US. Despite successful initial treatment with liposomal amphotericin B (LamB), relapse occurred. Treatments for VL in immunocompetent individuals are highly effective, but relapse can occur. There is more extensive experience in endemic areas with treating relapse that may be lacking in North America. This case alerts physicians in the US that immunocompetent adults can acquire VL during brief visits to endemic areas in Europe. It is important that travelers be counseled on preventive measures. Patients should be monitored after treatment for relapse.

## Case presentation

A 19-year-old college student with no significant medical history presented for evaluation of pancytopenia. He reported eight weeks of fatigue, sore throat, and neck adenopathy with progressive rigors, drenching night sweats, anorexia with 20-pound weight loss, and daily fevers of up to 103°F. Given worsening symptoms, he underwent additional testing that revealed pancytopenia and elevated transaminases. He was referred to a hematologist and was admitted for further evaluation and management of neutropenic fever.

One month before his symptoms began, he hiked in southern France (Department of Hérault) as well as Switzerland, Austria, Germany, Hungary, and Romania. Hérault extends from the city of Beziers to Montpelier along the south-central coast of France. The terrain is composed of low-lying coast in the south of the department and becomes rural, mountainous, and forested to the north and west. The patient did not encounter any stray animals or dogs during the trip and did not recall being bitten by insects. He did not use intravenous (IV) drugs or receive a blood transfusion while traveling. His sister, who accompanied him during the trip, was asymptomatic. He had traveled to an urban area in southwestern Guatemala six months before he became ill. Two years before presentation, he traveled to Mexico, Nicaragua, Croatia, Morocco, and Southeast Asia. He had no history of excessive alcohol intake or recreational drug use. He was not sexually active.

On admission, his temperature was 102.9°F (39.4°C), heart rate 120 beats per minute, and blood pressure 94/34 mmHg. He appeared pale with visible rigors. He had no scleral icterus, mucosal lesions, or lymphadenopathy. He had a nontender, enlarged spleen.

He had pancytopenia. Peripheral blood smear showed no blast forms or parasites. Liver transaminases, erythrocyte sedimentation rate (ESR), C-reactive protein (CRP), and ferritin were elevated. Blood cultures were sterile. Testing for HIV was negative. Laboratory studies are summarized in Table 1. Computed tomography scan of the chest, abdomen, and pelvis confirmed splenomegaly (19 cm) without lymphadenopathy.

**Table 1:** Summary of laboratory results.

Variable	Reference Range	Admission	Two Weeks Post Treatment	Relapse
**Hematology**				
WBC (cells/uL)	4–10 × 10^3^	2.0 × 10^3^	4.1 × 10^3^	1.8 × 10^3^
*Differential*:				
Neutrophils (%)	38–71	37	58.4	34
Lymphocytes (%)	14–46	45	31.6	36
Monocytes (%)	2–15	18	9.7	23
Hemoglobin (g/dL)	14–18	9.0	12.1	9.5
Platelets (× 10^3^ cells/uL)	150–350	117	234	69
ANC (× 10^3^ cells/uL)	1–9	0.7	2.4	0.7
MCV (fL)	78–94	83	90.2	85
Reticulocytes (%)	0.6–2.7	3.3	—	1.4
ESR (mm/h)	0–20	104	—	45
**Chemistry**				
AST (U/L)	0–30	99	78	37
ALT (U/L)	0–34	125	183	38
Alkaline phosphatase (U/L)	30–130	152	117	52
Total bilirubin (mg/dL)	0.2–1.1	0.53	0.6	0.16
Creatinine (mg/dL)	0.5–1.2	0.9	1.02	0.8
CRP (mg/L)	0.1–3.0	78.5	—	39.1
Ferritin (ng/mL)	18–370	2,700	—	551
**Microbiology and Immunology**				
*Leishmania* IgG (units)	0	15 (positive)	—	—
Immunochromatographic strip for detection of *Leishmania* antibodies in serum (CDC)	Not detected	*L*. *donovani infantum* detected	—	—
*Leishmania* PCR/DNA sequencing of bone marrow (CDC)	Not detected	*L*. *infantum/chagasi* detected	—	—
*Leishmania* culture (CDC)	Not detected	*L*. *infantum/chagasi* detected	—	—

**Abbreviations:** ALT, alanine transaminase; ANC, absolute neutrophil count; AST, aspartate transaminase; CDC, Centers for Disease Control; CRP, C-reactive protein; ESR, erythrocyte sedimentation rate; IgG, immunoglobulin G; MCV, mean corpuscular volume; PCR, polymerase chain reaction; WBC, white blood cell.

**Fig 1:**
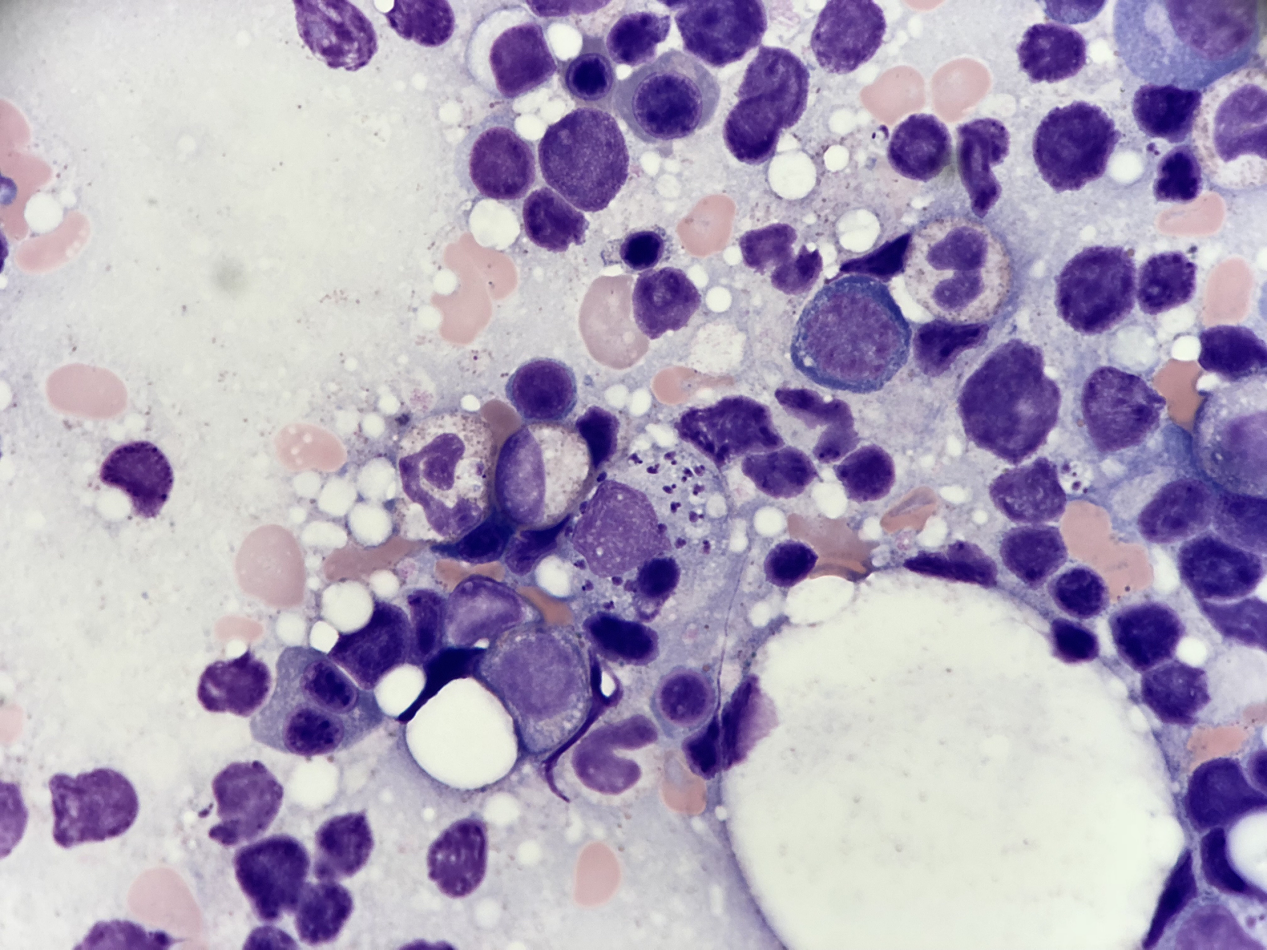
Bone marrow aspirate demonstrating *Leishmania* amastigotes. Bone marrow aspirate reveals macrophages with numerous nuclei of intracellular amastigotes and adjacent kinetoplasts of *Leishmania* parasites (Giemsa stain, 1000X).

Bone marrow evaluation revealed numerous small cytoplasmic amastigotes with kinetoplasts within tissue macrophages (histiocytes) by Giemsa stain (Fig 1). Morphologic findings were consistent with leishmaniasis. Serologic testing was positive for *Leishmania* immunoglobulin G (IgG). Visceral leishmaniasis (VL) was confirmed by the Centers for Disease Control (CDC) by histological review, culture, and PCR testing of the bone marrow aspirate; DNA sequencing identified *L*. *infantum*.

The patient was treated for five days with liposomal amphotericin B (LAmB; 3 mg/kg IV daily, followed by additional doses on days 14 and 21 [total 21 mg/kg]). His symptoms and splenomegaly resolved by the end of treatment. The patient was followed in the infectious diseases clinic and was monitored with serial complete blood counts (CBC) and liver chemistries (liver function test [LFT]). Pancytopenia resolved within two months (Table 1).

Three months after treatment, mild cytopenias developed without symptoms. After six months, the patient again developed fevers, weight loss, anorexia, and night sweats, prompting readmission. On exam, he was febrile with splenomegaly. His CBC showed pancytopenia; transaminases were mildly elevated (Table 1). Repeat bone marrow evaluation revealed intracellular microorganisms within macrophages consistent with *Leishmania* amastigotes. A PCR was not repeated due to high clinical suspicion for relapse. He was retreated arbitrarily with LAmB at an increased dose of 4mg/kg IV daily for five days followed by 10 mg/kg IV weekly for three weeks (total 50 mg/kg). His symptoms resolved at treatment completion. A third bone marrow evaluation done one week following the end of treatment showed no morphological evidence of leishmaniasis. Over one year later, he remains asymptomatic with resolution of splenomegaly, pancytopenia, and liver enzyme abnormalities.

## Discussion

*Leishmania* is a protozoan that exists as promastigotes extracellularly or as amastigotes in blood macrophages or tissue macrophages (histiocytes) [1]. Amastigotes appear elliptical on Giemsa staining. The kinetoplast, consisting of mitochondrial DNA, is directly adjacent to the nucleus of the amastigote and is a distinguishing feature.

*Leishmaniasis* is classically divided between Old and New World depending on location of acquisition. It can present in visceral, cutaneous, or mucosal forms. Old World leishmaniasis is acquired in the Eastern hemisphere, with VL caused by the *L*. *donovani* complex, species *L*. *infantum* or *L*. *donovani* [2–5]. *L*. *infantum* is endemic in the Mediterranean basin, sub-Saharan Africa, and the Middle East, whereas *L*. *donovani* exists in India and sub-Saharan Africa [3, 5]. In the New World (Latin America), VL can also be caused by the *L*. *donovani* complex, species *L*. *chagasi*, which is indistinguishable from *L*. *infantum* by morphology, PCR, and DNA sequencing [3, 5].

Along with anthroponotic *L*. *donovani* in Sudan and South Asia, zoonotic *L*. *infantum* found in canine reservoirs contributes to the approximately 50,000–90,000 annual cases of VL worldwide [4–6]. In the Cévennes mountains that encompass the French department of Hérault, 80% of canines are seropositive for *Leishmania* parasites using PCR [7]. After a female sand fly bite, the incubation period in humans varies from two weeks to over one year, and the severity of symptoms ranges from asymptomatic to life-threatening [4, 5]. Signs and symptoms including fevers, night sweats, weight loss, marked splenomegaly, and pancytopenia are hallmarks. In endemic areas, immunocompetent hosts develop progressive disease presumably after repeated exposures. However, because CD4+ type 1 helper T cells that produce interferon gamma have a critical role in activating macrophages, patients with HIV are at greater risk of developing VL [4, 5]. There is a case reported of a patient with acquired immune deficiency syndrome (AIDS) who developed VL after returning to the US after prior travel to multiple endemic areas and living in Greece for five years; however, given his underlying immune compromise, this was likely reactivation disease from an unspecified prior exposure [8].

It is often under-appreciated, particularly in the U.S., that immunocompetent adults such as our patient can acquire VL during brief visits to endemic areas such as the southern French departments of Alpes-Maritimes, Bouches-du-Rhône, and Hérault [9–11]. Only a few cases are described in the literature, including one case of a 73-year-old immunocompetent patient returning from Spain (after hiking the Camino de Santiago) to the US [12]. This scenario is well known in English, Dutch, and German travelers returning from endemic European locales (Spain, Italy, Greece, and Macedonia) [13–15]. As reported to the National Reference Center for Leishmaniases in France between 1999 and 2012, there were 268 autochthonous cases of VL, roughly 19 cases per year (range, 5–31) [9]. Of the 366 cases of VL (autochthonous and imported), 55.7% occurred in immunocompetent hosts (no risk factors such as HIV, organ transplantation, leukemia, solid organ cancer, or exposure to immunosuppressive therapy), and 44.9% of cases were in individuals aged 20 to 60 [9]. Our patient also traveled to Guatemala, which is endemic for *L*. *infantum/chagasi*; however, he stayed in an urban area where VL cases have not been reported [16]. He also visited an urban area in Morocco two years before; notably, reported cases of VL in Morocco are usually in infants and those with long-term exposure [17]. Therefore, we believe that exposure mostly likely occurred in France.

Left untreated, VL causes multiorgan failure and death secondary to infections and hemorrhage [3, 4]. LAmB is the drug of choice for treatment of VL [3, 18]. The drug has greater bioavailability, longer half-life, and improved macrophage uptake compared to alternatives [5]. Although treatment failure and relapse of VL is well described among patients with HIV coinfection, less is known about its management in immunocompetent hosts [3, 19, 20]. Despite adequate treatment, relapse of VL can occur in 5% to 10% of immunocompetent patients according to one retrospective cohort study in which LAmB was administered at 5 mg/kg IV daily for four days [20]. Initial doses of greater than 5 mg/kg IV may provide better entry of the medication into tissues, although various dosing regimens have been reported with similar efficacy [18]. Our patient had a robust initial clinical response with a regimen that is recommended in guidelines (3 mg/kg for 5 days then repeated at days 14 and 21) but nonetheless had a relapse [3]. Further evidence comparing LAmB treatment protocols in immunocompetent patients as well as identification of additional risk factors for relapse are necessary to guide alternative dosing regimens. Oral miltefosine has emerged as an alternative to LAmB in VL caused by *L*. *donovani* acquired in the Indian subcontinent. There are anecdotal reports of decreased efficacy in *L*. *infantum/chagasi* cases according to guidelines from the Infectious Diseases Society of America (IDSA) [21]. Clinical monitoring post treatment is recommended to gauge response [21]. In general, parasitological confirmation of response by repeat bone marrow evaluation is not recommended.

For prevention, it is paramount to avoid outdoor exposure during dusk to dawn hours, wear protective attire, and use N,N-diethyl-meta-toluamide (DEET)-containing insect repellant as well as permethrin-impregnated clothing [10].

## Conclusion

VL is a serious illness seen infrequently among immunocompetent travelers with brief exposures to endemic areas in Europe such as southern France. This is a rare case of imported VL from Europe in a traveler returning to the US. A detailed travel history is necessary to explain the diagnosis. Travelers to endemic areas in Europe who may have outdoor exposures require pretravel counseling with an emphasis on preventive measures. Treatments for VL in immunocompetent individuals are effective, but relapses may occur and should be managed with additional courses of LAmB.
